# A potential role for cannabichromene in modulating TRP channels during acute respiratory distress syndrome

**DOI:** 10.1186/s42238-021-00101-0

**Published:** 2021-10-01

**Authors:** Hesam Khodadadi, Évila Lopes Salles, Eunice Shin, Abbas Jarrahi, Vincenzo Costigliola, Pritesh Kumar, Jack C. Yu, John C. Morgan, David C. Hess, Kumar Vaibhav, Krishnan M. Dhandapani, Babak Baban

**Affiliations:** 1 Department of Oral Biology and Diagnostic Sciences, Dental College of Georgia, Augusta University, Augusta, GA USA; 2 Center for Excellence in Research, Scholarship and Innovation, Dental College of Georgia, Augusta University, Augusta, GA USA; 3 Medical College of Georgia, Augusta University, Augusta, GA USA; 4 Department of Neurosurgery, Medical College of Georgia, Augusta University, Augusta, GA USA; 5 European Medical Association (EMA), Brussels, Belgium; 6 Cannabinoid Research Program, Canadore College, North Bay, Ontario Canada; 7 Department of Surgery, Medical College of Georgia, Augusta University, Augusta, GA USA; 8 Parkinson’s Foundation Center of Excellence, Movement Disorders, Program, Department of Neurology, Medical College of Georgia, Augusta University, Augusta, GA USA; 9 Department of Neurology, Medical College of Georgia, Augusta University, Augusta, GA USA

**Keywords:** Cannabichromene, CBC, ARDS, TRPA1, TRPV1, COVID-19

## Abstract

**Background:**

Acute respiratory distress syndrome (ARDS) is a life-threatening clinical syndrome whose potential to become one of the most grievous challenges of the healthcare system evidenced by the COVID-19 pandemic. Considering the lack of target-specific treatment for ARDS, it is absolutely exigent to have an effective therapeutic modality to reduce hospitalization and mortality rate as well as to improve quality of life and outcomes for ARDS patients. ARDS is a systemic inflammatory disease starting with the pulmonary system and involves all other organs in a morbid bidirectional fashion. Mounting evidence including our findings supporting the notion that cannabinoids have potential to be targeted as regulatory therapeutic modalities in the treatment of inflammatory diseases. Therefore, it is plausible to test their capabilities as alternative therapies in the treatment of ARDS. In this study, we investigated the potential protective effects of cannabichromene (CBC) in an experimental model of ARDS.

**Methods:**

We used, for the first time, an inhalant CBC treatment as a potential therapeutic target in a murine model of ARDS-like symptoms. ARDS was induced by intranasal administration of Poly(I:C), a synthetic mismatched double-stranded RNA, into the C57BL/6 mice (6–10 male mice/group, including sham, placebo, and CBC treated), three once-daily doses followed by a daily dose of inhalant CBC or placebo for the period of 8 days starting the first dose 2 h after the second Poly(I:C) treatment. We employed histologic, immunohistochemistry, and flow cytometry methods to assess the findings. Statistical analysis was performed by using one way analysis of variance (ANOVA) followed by Newman–Keuls post hoc test to determine the differences among the means of all experimental groups and to establish significance (*p* < 0.05) among all groups.

**Results:**

Our data showed that CBC was able to reverse the hypoxia (increasing blood O_2_ saturation by 8%), ameliorate the symptoms of ARDS (reducing the pro-inflammatory cytokines by 50% in lung and blood), and protect the lung tissues from further destruction. Further analysis showed that CBC may wield its protective effects through transient receptor potential (TRP) cation channels, TRPA1 and TRPV1, increasing their expression by 5-folds in lung tissues compared to sham and untreated mice, re-establishing the homeostasis and immune balance.

**Conclusion:**

Our findings suggest that inhalant CBC may be an effective alternative therapeutic target in the treatment of ARDS. In addition, Increased expression of TRPs cation channels after CBC treatment proposes a novel role for TRPs (TRPA1 and TRPV2) as new potential mechanism to interpret the beneficial effects of CBC as well as other cannabinoids in the treatment of ARDS as well as other inflammatory diseases. Importantly, delivering CBC through an inhaler device is a translational model supporting the feasibility of trial with human subjects, authorizing further research.

## Introduction

Acute respiratory distress syndrome (ARDS) is a serious complication of sepsis, initiated by cytokine storm due to imbalance of host immunity with exaggerated inflammatory responses (Hu et al., 2020; Matthay et al., 2019). Despite controversy in the definition of ARDS, there is a consensus over the crucial role of Immune response dysregulation in the pathophysiology of ARDS (Matthay et al., 2019; Matthay et al., 2021). As a systemic inflammatory disease, ARDS is initiated by activation of alveolar macrophages, recruitment of neutrophils, and circulating macrophages to the stressed lung tissues. As a consequent of such cellular activations, ARDS advances with elevated level of pro-inflammatory cytokines such as IL-6, IL-17, and IFNγ, damaging lung tissues and eliciting accumulation of macromolecules manifested by edema and hypoxemia (Han & Mallampalli, 2015). Contrary to all advancements in understanding the mechanisms and pathophysiology of ARDS, however, no pharmacologic modality for the treatment of ARDS has been yet identified. As evidenced by the COVID-19 pandemic, supportive therapies along with combinatorial therapies such as corticosteroid and IL-6 inhibitor have shown beneficial effects in the management of ARDS (Pan et al., 2018; Harahwa et al., 2020; Catherine, 2014). Therefore, the need to develop alternative novel therapeutic targets for the prevention and treatment of ARDS persists.

Cannabinoids are naturally occurring compounds in *Cannabis* plants (Atakan, 2012)*.* Numerous studies suggest beneficial effects of cannabinoids in clinical settings (Kogan, 2007; The Health Effects of Cannabis and Cannabinoids: The Current State of Evidence and Recommendations for Research, 2017; Maione et al., 2011). Of over 100 known cannabinoids, *four*, including tetrahydrocannabinol (*Δ*^9^-THC), cannabidiol (CBD), cannabinol (CBN), and cannabichromene (CBC), have attracted the most attention due to supporting evidence of their potential as therapeutic targets (Maione et al., 2011; Izzo et al., 2012; Patil et al., 2020; Martin et al., 2021.; Anderson et al., 2021). Despite their structural differences, both CBD and CBC are non-psychoactive compounds with potential immunomodulatory effects and several other health benefits (Maione et al., 2011; Izzo et al., 2012; Pollastro et al., 2018). More recently, in a set of preclinical studies, we showed for the first time that CBD could ameliorate the symptoms of ARDS (Salles et al., 2020; Khodadadi et al., 2020). Importantly, unlike THC and CBD, CBC is not categorized as a scheduled compound by US Drug Enforcement Agency (DEA), which may facilitate the use and assessment of CBC in clinical settings (BayMedica White paper on Cannabichromene, n.d.). Further, several reports suggest that CBC may act through the transient receptor potential (TRP) cation channel subfamily A member 1 (TRPA1) as well as transient receptor potential cation channel subfamily V member 1 (TRPV1) with less affinity. TRPs are involved in vast variety of vital cellular processes, influencing the dynamic of physiologic and immunologic interactions within the multicellular organism (Muller et al., 2019; Froghi et al., 2021; Kun et al., 2014; Tsumura et al., 2012; Romano et al., 2013). CBC binding to TRPs would likely affect tissue homeostasis and immune balance, modulating the inflammatory responses as well as endocannabinoid cellular reuptake (Pollastro et al., 2018; De Petrocellis et al., 2011). CBC has also been reported to be an agonist to the CB2 receptor, activation of which may further modulate inflammatory responses (Izzo et al., 2012; Udoh et al., 2019).

In this study, we investigated the potential protective effects of CBC, possible mechanisms of action, and whether CBC can improve symptoms in an experimental model of ARDS.

## Materials and methods

### Animals

The studies utilized male, 10–12 weeks of age, C57BL/6 mice which were obtained from Jackson Laboratory USA and housed in the laboratory animal facilities of the Augusta University with free access to food and water. These studies conformed to guidelines of Institutional Animal Care and Use Committee.

### Experimental design

**Fig. 1:**
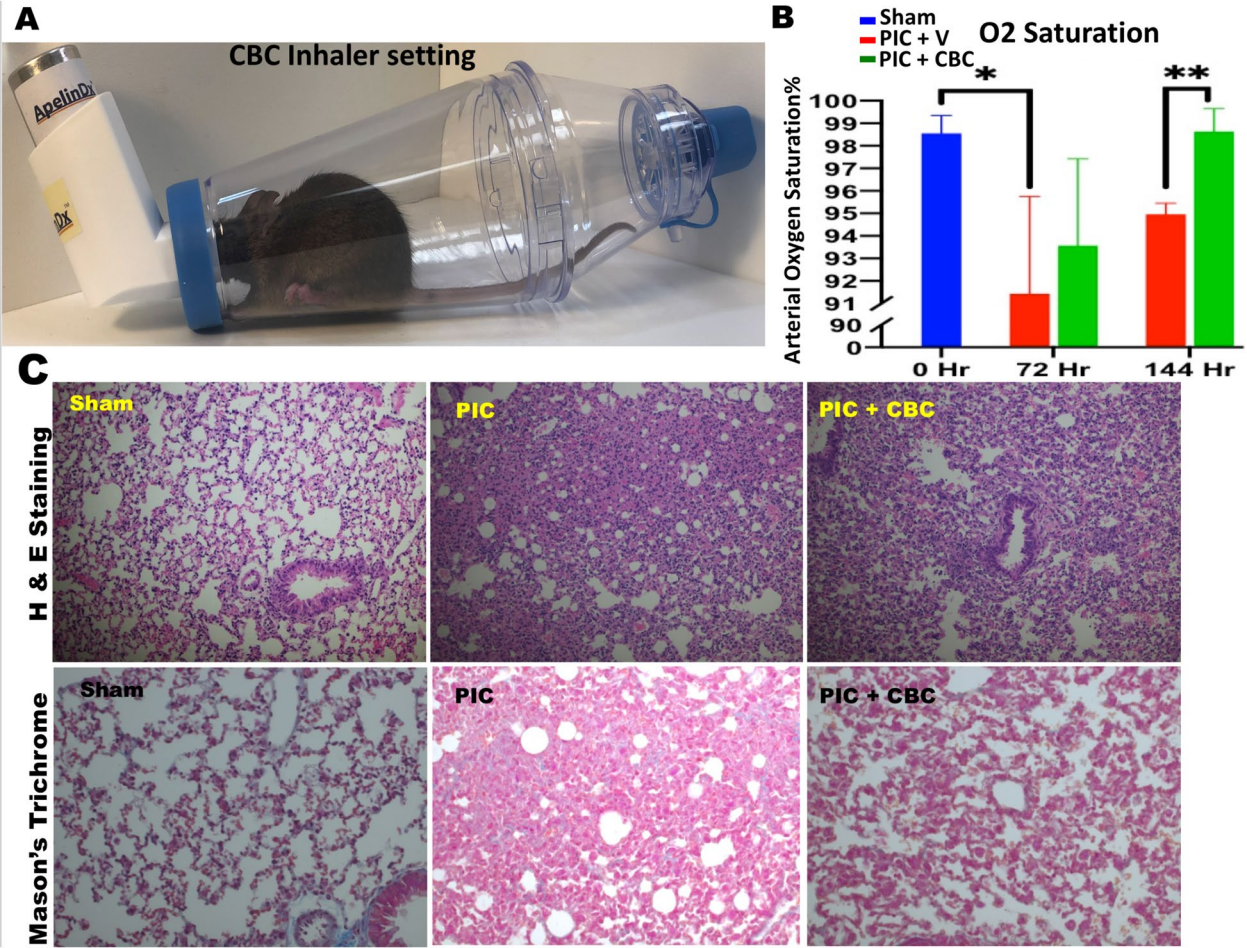
Inhalant cannabichromene (CBC) improved pulmonary function. **A** Treating mouse model of acute respiratory distress syndrome (ARDS) with inhalant CBC, specifically designed cone-shaped spout of the inhaling device (ApelinDx^tm^) connected to spacer completely sealed for a soft, comfortable, and insuring delivery of inhalant CBC to the mice. **B** Blood oxygen saturation was increased by ~8% towards a normal level (Blue bar) in mice treated with Poly (I:C) (PIC) followed by the treatment of inhalant CBC (PIC + CBC) (Green bar) compared to the untreated group (PIC) (Red bar) (**p<0.05, bars show means +/− standard deviation, SD). **C** Histological analysis, hematoxylin and eosin (H&E upper panels), and Masson’s trichrome (MT lower panels) demonstrated that Poly (I:C) caused the destruction of morphology and architecture of lung in mice with ARDS compared to the normal control group (sham). Inhalant CBC was able to improve the lung structure towards a normal status (PIC + CBC)

ARDS was induced as described previously (Salles et al., 2020; Khodadadi et al., 2020). Briefly, mice were divided into three groups: sham, control, and treatment (*n* = 6–10) in an open-labeled fashion to explore the efficacy of the treatment. All experiments were performed in accordance with the rules and regulations of the Augusta University Institutional Animal Care and Use Committee (IACUC). All mice were anesthetized with isoflurane. Sham group received phosphate-buffered saline (PBS) while control and treatment groups were administered Poly(I:C) (Sigma Aldrich, St. Louis, MO, USA) (100 mg in 50 mL sterile PBS) intranasally (IN) three once-daily doses. CBC was generously provided by BayMedica USA (Prodiol^TM^ CBC) and was administered through an inhaler (ApelinDx^tm^, TM Global Bioscience) (5 mg/kg, Fig. 1A), first dose 2 h after the second Poly(I:C) treatment, and every other day for a total of three doses to the treatment group. The dose and administration route of the drug were determined based on previous independent as well as our own studies (Khodadadi et al., 2020; Millar et al., 2019; Borghardt et al., 2018; Rau, 2005). Inhalant delivery provides advantages including local drug delivery with expected minimal side effects, rapid onset and high efficiency impact on the target site and simulating common route of administration seen in clinical scenarios. Sham and control groups received placebo through inhaler carrying the vehicle only. All mice were sacrificed at 8 days after the first Poly(I:C) application. Blood and lung tissues were harvested and subjected to further analysis. Blood oxygen saturation was measured using portable pulse oximeter through carotid arteries, before and after any treatment.

### Histology and immunohistochemistry

Multiple 5 μm midcoronal paraffin-embedded sections of lung tissues were cut and stained with hematoxylin and eosin, or trichrome. As for inflammatory indices, immunohistochemistry was performed as described previously (Salles et al., 2020; Khodadadi et al., 2020). Briefly, paraffin sections of lung tissues were deparaffinized in xylene and rehydrated by passing the slides through graded alcohol solutions. Endogenous peroxidase was quenched with 3% H_2_O_2_ in PBS. The sections were then washed in distilled water and heated at 95 °C in antigen retrieval buffer (Dako, Glostrup, Denmark). Nonspecific staining was blocked with 5% normal goat serum in PBS for 1 h. Endogenous biotin was inhibited with an avidin/biotin blocking kit (SP2001, Vector Laboratories, Burlingame, CA). The sections were then incubated with anti-murine TRPA1, and TRPV1. Preparations were counterstained with hematoxylin and mounted in Faramount and imaged by bright field microscopy. The expression of TRPA1 and TRPV1 were quantified by using particle count and color intensity measurement in the ImageJ software version 1.53.

### Flow cytometry analyses

For analytical flow cytometry, single-cell suspension was prepared from lung and blood tissues as described previously (Salles et al., 2020; Khodadadi et al., 2020). In brief, tissue samples were sieved through a 100 μM cell strainer (BD Biosciences, San Diego, CA, USA), followed by centrifugation (252 g, 10 min) to prepare single-cell suspensions. Then cells were fixed and permeabilized and stained intracellularly for cytokines including IL-6, IL-17, and interferon gamma (IFNγ) (proinflammatory cytokines). All samples were run through a 4-Laser LSR II flow cytometer. Cells were gated based on forward and side scatter properties and on marker combinations to select cells of interest. All acquired flow cytometry data were analyzed using FlowJo V10. Graphs and summary statistics were also used to assess the results.

### Statistics

In order to determine the statistical differences among the means of all experimental groups, data were analyzed using one way analysis of variance (ANOVA) followed by Newman–Keuls post hoc test for multiple comparison and to establish significance (*p* < 0.05) among all groups.

## Results

**Fig. 2:**
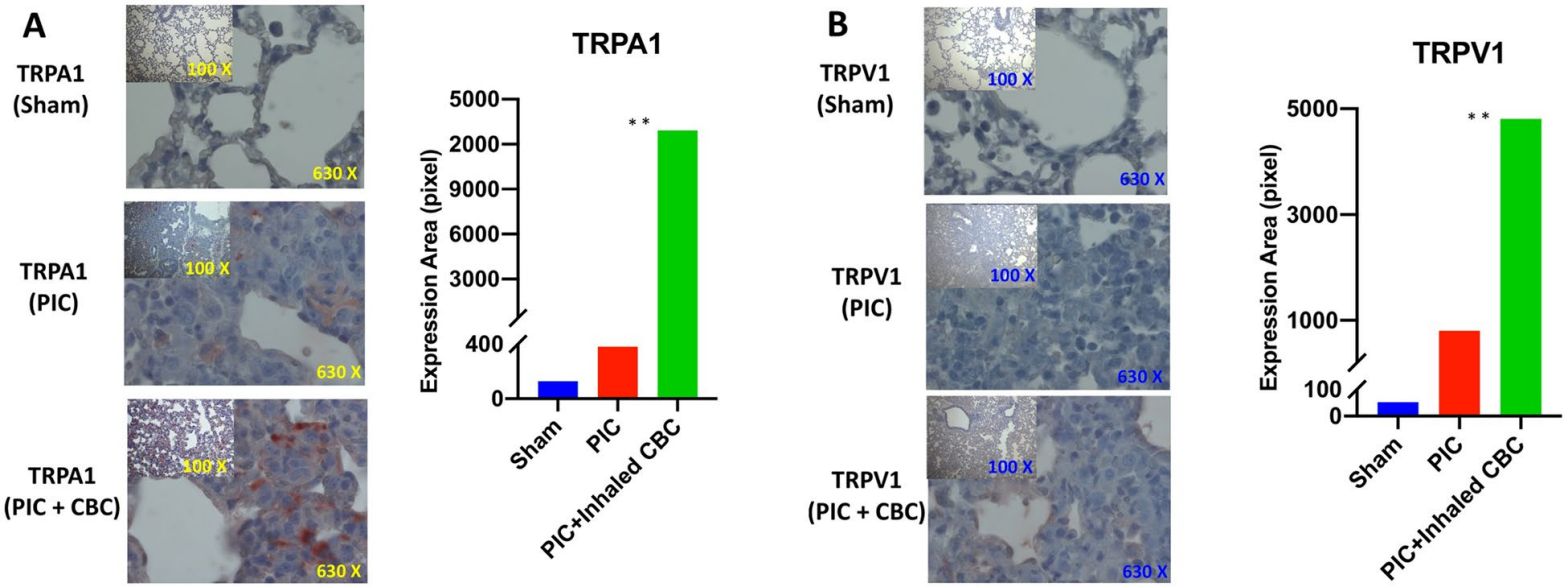
Inhalant cannabichromene (CBC) may interact with the endocannabinoid system through enhancement of transient receptor potential (TRP) cation channels. Immunohistochemical staining of lung tissues with **A** transient receptor potential cation channel subfamily A member 1 (TRPA1) and **B** the transient receptor potential cation channel subfamily V member 1 (TRPV1) with less affinity showed an increase in the expression level of TRPA1 and TRPV1 in lung tissues of CBC treated group (PIC + CBC) compared to normal sham or Poly (I:C) treated one (sham or PIC) (images are all ×630 and inner images are ×100 magnification) (**p<0.01, means +/-SD). The bar graphs demonstrate the quantified analysis of the staining using particle count and color intensity measurement in the ImageJ software version 1.53

**Fig. 3:**
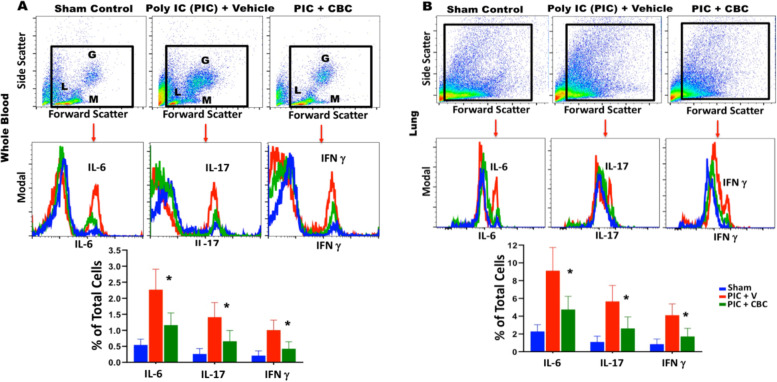
Inhalant cannabichromene (CBC) reduced ARDS-like induced inflammatory indices. Flow cytometry analysis of **A** whole blood and **B** lung tissues showed that Intranasal administration of Poly(I:C) effectuated a significant increase of pro-inflammatory cytokines including IL-6, IL-17, and IFNγ (PIC group) compared to the control group (sham). These effects were reversed with inhalant CBC (PIC + CBC), resulted in marked downregulation of all three pro-inflammatory cytokines (*p<0.03, means +/− SD). Further, the flow cytometry analysis of whole blood in the PIC group showed increased granulocytes (G) and monocytes (M) while lymphocytes were decreased compared to mice treated with inhalant CBC (PIC + CBC) supporting the notion that inhalant CBC can reverse the adverse effects of ARDS and re-establish the immune balance, resolving lymphopenia and restoring homeostasis

Inhalant CBC reversed hypoxia, increasing blood oxygen saturation positively towards the normal level by 8% from 90% to 98% (+/− 0.5%) (Fig. 1A, B). Histological examination of lung tissues, hematoxylin and eosin staining (Fig. 1C upper panels), and Masson’s trichrome staining (Fig. 1C lower panels) demonstrated that CBC treatment reduced the ARDS-like lung destruction, improved the structural damages to the lung. Further histological examination, immunohistochemistry showed that CBC treatment increased the level of expression for transient receptor potential (TRP) channels of ankyrin type-1 (TRPA1) and vanilloid type-1 (TRPV1), suggesting an active interaction between CBC and TRPs (Fig. 2). Flow cytometry analysis of blood and lung demonstrated that CBC treatment curtailed the expression levels of pro-inflammatory cytokines including IL-6, IL-17, and IFNγ in both tissues compared to the untreated counterparts (*p* < 0.01) (Fig. 3A, B). The histograms show the quantified level of expression of IL-6, IL-17, and IFNγ in both tissues treated with CBC compared to the control sham (untreated with CBC) group.

## Discussion

Our findings demonstrated, for the first time, that inhalant CBC protected lung structure, contained excessive cytokine production, and curtailed inflammatory responses both in lung and blood tissues in an experimental ARDS model. These results were in consistent with findings by other independent studies demonstrating counter-inflammatory effects of CBC in LPS-induced paw edema (DeLong et al., 2010). In addition, CBC is a non-psychoactive cannabinoid which is not categorized as a scheduled compound by US Drug Enforcement Agency (DEA). This may facilitate the use and assessment of CBC in clinical settings (BayMedica White paper on Cannabichromene, n.d.). More importantly, using inhalers for the first time in a therapeutic setting would provide an effective and safer way to deliver CBC to small airways, lowering required doses and reducing any potential systemic adversarial symptom in a more cost-effective way. Additionally, delivery of CBC through an inhaler increases the translational values of this study and potential for trials in a clinical setting with implications for the treatment of respiratory complications such as development of ARDS in severe cases of COVID-19 and several respiratory diseases. It is noteworthy that we exclusively employed male animals in our studies to prevent additional confounding factors based on gender differences (e.g., different stages of estrous cycle, hormonal effects), staying focused on the proof of concept for CBC sole effects in ARDS. However, increasing evidence suggests a crucial role for sex dimorphism in occurrence and outcomes of ARDS, warranting further investigations at the pre-clinical level as well as for the potential human clinical trials in the future (Scully et al., 2020).

Further, our data showed that CBC can increase the expression level of transient receptor potential ankyrin 1 (TRPA1) and vanilloid receptor 1 (TRPV1), supporting the notion of potential interactions between CBC and TRPA1 as well as TRPV1 (TRPs). The TRP channels superfamily play vital roles in a wide range of physiological and immunologic processes such as cellular polarization, cytokine production, phagocytosis, and cytotoxicity (Muller et al., 2019; Froghi et al., 2021; Kun et al., 2014; Tsumura et al., 2012). Therefore, it is very plausible to hypothesize that binding CBC to TRPs may downregulate the inflammatory responses by shifting the so-called “cytokine storm” to a “cytokine breeze,” re-establishing the homeostasis and preventing additional structural damages to the vital organs. Although the interaction between CBC and TRPs has been previously reported (Maione et al., 2011; De Petrocellis et al., 2011), to the best of our knowledge, this is the first report to show the beneficial effects of cross-talk between CBC and TRPs in ARDS. In addition, since several studies have indicated that CBC may bind to and activate CB2 receptor of the endocannabinoid system, suggesting a potential synergy between TRPs and CB2 activation as a potential mechanism contributing to the beneficial effects of CBC in ARDS. In fact, such potential engagement of the endocannabinoid system by CBC may establish a synergistic relationship between exogenous phytocannabinoids (CBC) and endogenous endocannabinoids (e.g., anandamide and 2-AG). Such bi-directional dynamic would have the potential as a therapeutic modality in the treatment of ARDS as well as a wide variety of inflammatory diseases, neurodegenerative diseases, pain control, cancer, chronic wounds, and sleeping disorders.

## Conclusion

These novel findings render a new therapeutic role for CBC in the treatment of ARDS as well as a wide range of respiratory diseases including a potential for COVID-19 and other inflammatory conditions. The use of inhalant CBC and direct delivery of CBC to the lungs offers a more rapid onset of action, allows smaller doses to be used, and has a better efficacy to safety ratio compared to systemic therapy. Further, as TRPs are emerging as novel therapeutic targets through their potential roles in cellular interactions and tissue homeostasis (Zhao et al., 2021), therefore, it is plausible to envision more prominent roles for CBC in tissue homeostasis, modulation of inflammatory responses, and orchestrating a set of cellular interactions as an immunotherapeutic target, warranting further research.

## Data Availability

The datasets used and/or analyzed during the current study are available from the corresponding author on reasonable request.

## Abbreviations

ARDS: Acute respiratory distress syndrome
CBC: Cannabichromene
CBD: Cannabidiol
CBN: Cannabinol
DEA: Drug Enforcement Agency
IL-6: Interleukin-6
IL-17: Interleukin-17
IFNγ: Interferon gamma
LPS: Lipopolysaccharides
PIC: Poly I:C
PBS: Phosphate-buffered saline
TRP: Transient receptor potential
TRPA1: Transient receptor potential ankyrin 1
TRPV1: Transient receptor potential vanilloid receptor 1
2-AG: 2-Arachidonoylglycerol
Δ9-THC: Tetrahydrocannabinol
